# Computed tomographic analysis of the quality of trunk muscles in asymptomatic and symptomatic lumbar discectomy patients

**DOI:** 10.1186/1471-2474-12-65

**Published:** 2011-03-31

**Authors:** Katie GW Bouche, Olivier Vanovermeire, Veerle K Stevens, Pascal L Coorevits, Jacques J Caemaert, Dirk C Cambier, Koenraad Verstraete, Guy G Vanderstraeten, Lieven A Danneels

**Affiliations:** 1 Department of Physical Medicine and Rehabilitation, Faculty of Medicine and Health Sciences, Ghent Universitary Hospital, De Pintelaan 185, 9000 Ghent, Belgium; 2 Department of Radiology, AZ Groeninge, Loofstraat 43, 8500 Kortrijk, Belgium; 3 Department of Traumatology & Rehabilitation, Section Evaluation, Prevention, Research & Development, Military Hospital of Base Queen Astrid, Belgian Ministry of Defense, Bruynstraat 2, Brussels, Belgium; 4 Department of Public Health & Research in Advanced Medical Informatics and Telematics, Faculty of Medicine and Health Sciences, Ghent University Hospital, De Pintelaan 185, 9000 Ghent, Belgium; 5 Department of Neurosurgery, Faculty of Medicine and Health Sciences, Ghent Universitary Hospital; De Pintelaan 185, 9000 Ghent, Belgium; 6 Department of Rehabilitation Sciences and Physical Therapy, Faculty of Medicine and Health Sciences, Ghent Universitary; Belgium Universitary; Belgium; 7 Deârtment of Radiology, Faculty of Medicine and Health Sciences, Ghent Universitary Hospital; De Pintelaan 185, 9000 Ghent, Belgium

## Abstract

**Background:**

No consensus exists on how rehabilitation programs for lumbar discectomy patients with persistent complaints after surgery should be composed. A better understanding of normal and abnormal postoperative trunk muscle condition might help direct the treatment goals.

**Methods:**

A three-dimensional CT scan of the lumbar spine was obtained in 18 symptomatic and 18 asymptomatic patients who had undergone a lumbar discectomy 42 months to 83 months (median 63 months) previously. The psoas muscle (PS), the paraspinal muscle mass (PA) and the multifidus muscle (MF) were outlined at the L3, L4 and L5 level. Of these muscles, fat free Cross Sectional Area (CSA) and fat CSA were determined. CSA of the lumbar erector spinae (LES = longissimus thoracis + iliocostalis lumborum) was calculated by subtracting MF CSA from PA CSA. Mean muscle CSA of the left and right sides was calculated at each level. To normalize the data for interpersonal comparison, the mean CSA was divided by the CSA of the L3 vertebral body (mCSA = normalized fat-free muscle CSA; fCSA = normalized fat CSA). Differences in CSA between the pain group and the pain free group were examined using a General Linear Model (GLM). Three levels were examined to investigate the possible role of the level of operation.

**Results:**

In lumbar discectomy patients with pain, the mCSA of the MF was significantly smaller than in pain-free subjects (*p *= 0.009) independently of the level. The mCSA of the LES was significantly smaller in pain patients, but only on the L3 slice (p = 0.018). No significant difference in mCSA of the PS was found between pain patients and pain-free patients (p = 0.462). The fCSA of the MF (*p *= 0.186) and of the LES (p = 0.256) were not significantly different between both populations. However, the fCSA of the PS was significantly larger in pain patients than in pain-free patients. (p = 0.012).

The level of operation was never a significant factor.

**Conclusions:**

CT comparison of MF, LES and PS muscle condition between lumbar discectomy patients without pain and patients with protracted postoperative pain showed a smaller fat-free muscle CSA of the MF at all levels examined, a smaller fat- free muscle CSA of the LES at the L3 level, and more fat in the PS in patients with pain. The level of operation was not found to be of importance. The present results suggest a general lumbar muscle dysfunction in the pain group, in particular of the deep stabilizing muscle system.

## Background

Following lumbar discectomy, residual complaints persist to some degree in 28% [1] to 74.6% [2] of patients and are a common diagnostic and therapeutic problem. Previous studies have focused on the radiological identification of possible pain-inducing structures in failed back surgery patients [3-5]. However, recurrent pain following lumbar surgery is clinically often nonspecific, and imaging techniques frequently fail to demonstrate a structural reason for the pain. As a consequence, no consensus exists on the management of such residual pain, especially if technical investigations are negative. Exercise therapy following surgery has been shown to have a beneficial effect [6-8], but how rehabilitation programs should be composed remains a controversial issue [7].

As in nonspecific chronic low back pain (LBP) [9-11], the paraspinal muscles seem atrophied in patients with postoperative LBP [12-14]. Postoperative trunk extensor atrophy has been shown to be accompanied by a decrease in muscle function, particularly in trunk extension force [13]. The most medial of the three paraspinal muscles (PA), the multifidus (MF), has a major trunk-stabilizing function [5,15]. In nonsurgical LBP patients, MF atrophy has been demonstrated, and current physiotherapy practice is often focused on localized spine-stabilizing muscle exercises [16,17].

Previous studies have reported on the Computed Tomography (CT) quality of the back muscles of lumbar discectomy patients [12-14]_. _Muscle atrophy has been scored on CT-images of patients with good and poor recovery 2 to 5 years after surgery for spinal stenosis or disc herniation [14]. In the study by Sihvonen et al, distinct back muscle atrophy was much more prevalent in patients with poor results. Muscle disuse was held responsible for this finding, because muscle atrophy was not restricted to the level of operation in the failed back group [14]. The rating of muscle atrophy was, however, partially based on a visual impression of back muscle density, without specification of muscle mass. Cooper et al. demonstrated simultaneous wasting of the PA and the psoas (PS) in chronic LBP (mainly surgical patients) compared to acute LBP patients [12]. The MF was not studied separately [12]. Mayer et al. described PS and erector spinae atrophy in spinal surgery patients (27 mechanical/chemical discectomy patients and 7 lumbar fusion patients) compared to controls without back pain. Muscle atrophy was documented through a significant decrease in muscle density on CT scan 3 months after surgery [2]. Whether or not patients experienced postoperative pain was however not taken into account. The MF - which is retrected in standard lumbar discectomy and in lumbar fusion - was not investigated separately. Motosuneya et al. studied back muscle atrophy on MRI images after five surgical procedures [18]. They found significant back muscle atrophy after anterior lumbar interbody fusion, posterior lumbar interbody fusion and posterolateral fusion, but not after laminectomy and nucleotomy. The MF was not investigated separately, and most patients had no or occasional mild LBP.

To our knowledge, differences in muscle quality of the isolated MF between lumbar discectomy patients with and without pain have not been studied yet.

Therefore, the present study was designed to investigate possible differences in muscle condition of 3 trunk muscles, particularly the MF, in lumbar discectomy patients with pain and without pain. The muscles were examined at three levels to study the possible influence of the level of operation.

## Methods

### Study design

After obtaining approval from the Ghent University Ethics Committee, lumbar discectomy patients with and without pain were included in the study. A volume CT scan of the lumbar region (L3 lower endplate to S1 lower endplate) was performed to screen for old and new lumbar disorders. In case of normal postoperative findings, reconstructions were made through the lower endplates of L3, L4 and L5 for measurement of the total and fat-free muscle cross-sectional area (CSA) of the isolated MF, lumbar erector spinae (LES) and PS on both the left and right side. Fat area was calculated as the subtraction of fat-free muscle CSA from total CSA.

### Subjects

Thirty-six patients with a history of L5-S1 lumbar discectomy, participated in the study. They were divided into a pain-free postdiscectomy group (n = 18) and a postdiscectomy group with pain (n = 18), based on their pain history and the results of a Visual Analogue Scale (VAS) for pain. Pain-free patients had experienced no or occasional back pain following the operation. The cut-off point was set at 1.5 on VAS since 7 patients scored between 0.5 and 1.5 on VAS, stating that this score reflected no real pain, but rather awareness of their back [19].

Disc resection was unilateral in 20 (56%) and bilateral in 16 (44%) cases. Time since surgery ranged from 12 months to 89 months (mean 59 months). All participants read and signed an informed consent form. Their clinical data are presented in table 1.

**Table 1:** Questionnaire scores and clinical characteristics of the lumbar discectomy patients

		Discectomy, no pain	Discectomy with pain	*P-*value
N		18	18	
Gender	male	8	8	
	female	10	10	
Age (yrs)	Mean (SD)	44.39 (± 9.27)	49.72 (± 9.33)	0.094
BMI^1 ^((%)	Mean (SD)	25.74 (± 3.36)	26.02 (± 3.36)	0.807
Pain on VAS^2 ^(cm)	Mean (SD)	0.69 (± 0.65)	4.7 (± 1.5)	<0.001
Radicular pain			11	
Low back pain			7	
Time since surgery (months)	Mean (SD)	56.00 (± 18.72)	61.50 (± 14.40)	0.405
Duration pain before operation (months)	Mean (SD)	57.06 (± 78.25)	62.12 (± 58.09)	0.427
motor	No paresis	16	13	
	paresis	2	5	
sensibility	Normal	12	12	
	Abnormal	6	6	
Reflexes	Normal	15	12	
	Abnormal	3	6	
Lasègue	Positive	0	3	
	Negative	18	15	
Kemp	Positive	1	5	
	Negative	17	13	
Slump	Positive	3	5	
	Negative	15	13	
QBPDS ^3^	Mean (SD) Min-max	14.41 (± 10.81) 0-36	41.72 (± 18.18) 16-75	<0.001
MPI ^4^	Mean (SD) Min-max	45.71(± 16.12) 17-83	75.69 (± 20.68) 45-108	<0.001
TAMPA	Mean (SD) Min-max	33.50 (± 9.71) 10-47	39.56 (± 6.33) 27-49	0.041

^1^Body Mass Index; ^2 ^Visual Analogue Scale; ^3 ^Quebec Back Pain Disability Scale; ^4^Multidimensional Pain Inventory

#### Exclusion criteria

Overall exclusion criteria were lumbar scoliosis, hip disorders, pregnancy within the last year, a history of central neurological impairments, and major pathological conditions such as a malignant tumour or uncontrolled systemic disease. Patients with abnormal postoperative CT-findings were also excluded from the study. These abnormal findings included recurrent disc herniation or new disc herniation with compression of the spinal cord or nerve root compression, spinal stenosis, spondylolisthesis, spinal tumour or pseudomeningomyelocele, degenerative narrowing of the lateral recess with compression of neural structures, and stress fracture of the vertebral arch.

### Procedure

#### Interview, clinical examination, questionnaires

Clinical evaluation of outcome included an interview and a neurological examination by an independent observer, and standard questionnaires (Quebec Back Pain Disability Questionnaire (QBPDS)[20-22], VAS for pain, Multidimensional Pain Inventory Part I (MPI) [23,24] and TAMPA scale for kinesiophobia) [25].

#### Computed tomography

**Figure 1:**
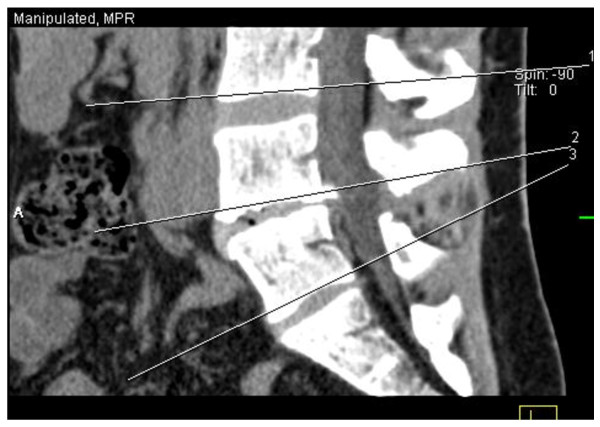
Sagittal view representing the three standardized slices along the lower end-plate of L3, L4 and L5 vertebrae

A 4-slice CT-scan (Somatom Volume Zoom, Siemens Medical Systems, Germany) was used at 140 kV and 200 mAs with a slice collimation of 1.25 mm and a pitch of 0.75. The patient was scanned in prone position from the lower endplate of the L3 to the S1 vertebral body, without contrast administration. Adjacent 5-mm reconstructions were made in a soft-tissue kernel (B30s, medium smooth). All scans were evaluated on a Leonardo (Siemens Medical Solutions, Germany) workstation by a single radiologist, blinded for the patient's complaints. Five-mm thickness reconstructions were made through the lower endplate of the L3, L4 and L 5 vertebral bodies in a standard fashion (figure 1).

**Figure 2:**
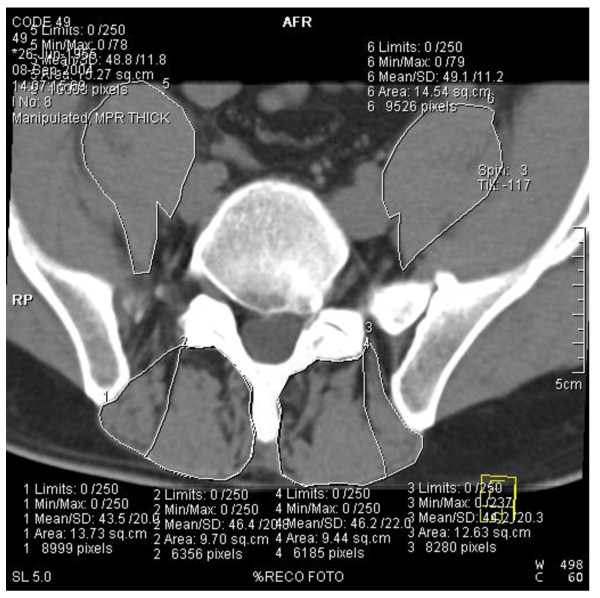
**Illustration of the boundaries of the Region of Interest (ROI) of the psoas, paravertebral mass and multifidus**. Computed tomographic scan at L5 level 76 months after bilateral L5-S1 discectomy in a 49-year old man with pain.

At the L3, L4 and L5 levels, a Region of Interest (ROI) was set at the borders of the PS (psoas major + iliac muscle), the PA (PA = MF + erector spinae: posterior border of the vertebra, dorsal border of MF and dorsolateral border of LES) and the isolated MF (following its lateral fascia where possible and if not, taking the middle of the intermuscular area between the MF and the LES) (figure 2).

The total CSA (fat+muscle) of the different muscles was measured. Next, fat-free muscle CSA was determined using a threshold technique (Osiris), in which the area occupied by pixels within the soft-tissue threshold limits (0 -250 HU) was selected. Fat CSA was calculated as the difference between total CSA and fat-free muscle CSA. The CSA of the LES was calculated by subtracting the MF CSA from the PA CSA. The CSA of the L3 vertebral body was measured to normalize the data: each CSA was divided by the CSA of this body, resulting in normalized fat-free muscle CSA data (mCSA) and normalized fat CSA (fCSA) data.

### CT Measurement Reliability

All measurements were performed by the same examiner. Intratester reliability was assessed by repeated measurement of 20 of the 36 scans. The assessment of measurement repeatability showed good agreement between the two measurements for PS (ICC = 0.92), LES (ICC = 0.97) and MF (ICC = 0.96), indicating that the measures were reliable.

### Data Analysis

Descriptive statistics were used to analyse patient characteristics and clinical findings.

Mann-Whitney-U tests were performed to investigate differences in patient group characteristics. For each muscle investigated (MF, LES, PS) a repeated measures analysis of variance with 3 factors was performed to investigate possible differences in mCSA and fCSA between pain-free and pain patients. The between subject factors were 'group' (with 2 sublevels: pain and no pain) and 'level of operation' (with 2 sublevels: L4 discectomy and L5 discectomy). The within subject factor was 'slice' (L3 slice/L4 slice/L5 slice).

The statistical analyses were performed with SPSS 15.0 software (SPSS, Chicago, IL). Statistical significance was set at *P *< 0.05.

## Results

The asymptomatic and symptomatic lumbar discectomy group did not significantly differ in age, gender, Body Mass Index (BMI), duration of pain before surgery and time elapsed since surgery (table 1). QBPDS (*p *< 0.001), MPI (*p *< 0.001) and TAMPA (p = 0.039) outcome scores were significantly higher in the pain group.

For both groups, the results of the mCSA and fCSA measurements of the LES, MF and PS at the three levels, and of the CSA of the L3 lower endplate are shown in table 2.

**Table 2:** L3 lower endplate CSA, fat-free muscle CSA (mean of left and right side), intramuscular fat area (mean of left and right side), and mean normalized fat free muscle CSA of the psoas (PS), lumbar erector spinae (LES) and multifidus (MF) in lumbar discectomy patients with and without pain

	Section		Muscle	Discectomy, no pain	Discectomy with pain
Bony CSA	L3 lower endplate	cm^2^		15,98 ± 2.70	15.30 ± 2.65

Fat-free muscle CSA	L3 lower endplate	Cm^2^	PS	21.37 ± 6.65	18.71 ± 5.56
			LES	34.57 ± 8.36	28.62 ± 6.31
			MF	13.25 ± 3.38	10.95 ± 3.44
	L4 lower endplate		PS	±	±
			LES	25.69 ± 7.16	21.79 ± 5.06
			MF	17.22 ± 3.59	14.38 ± 3.97
	L5 lower endplate		PS	±	±
			LES	14.17 ± 6.31	10,78 ± 5.03
			MF	19.49 ± 3.79	15.56 ± 3.32

Normalized fat-free	L3 lower endplate		PS	1.33 ± 0.29	1.21 ± 0.24
muscle CSA			LES	2.21 ± 0.44	1.87 ± 0.34
			MF	0.83 ± 0.17	0.71 ± 0.16
	L4 lower endplate		PS	±	±
			LES	1.64 ± 0.39	1.44 ± 0.32
			MF	1.08 ± 0.18	0.94 ± 0.20
	L5 lower endplate		PS	±	±
			LES	0.90 ± 0.43	0.72 ± 0.30
			MF	1.23 ± 0.21	1.02 ± 0.15

Fat area	L3 lower endplate	cm^2^	PS	0.73 ± 0.45	0.91 ± 0.53
			LES	3.26 ± 1.28	4.34 ± 2.13
			MF	2.52 ± 1.16	3.52 ± 1.84
	L4 lower endplate		PS	±	±
			LES	1.21 ± 0.80	1.54 ± 0.87
			MF	3.65 ± 1.61	5.08 ± 2.46
	L5 lower endplate		PS	±	±
			LES	3.56 ± 1.43	5.22 ± 3.05
			MF	5.41 ± 1.87	7.19 ± 3.00

Normalized fat CSA	L3 lower endplate		PS	0.05 ± 0.03	0.06 ± 0.03
			LES	0.22 ± 0.09	0.29 ± 0.17
			MF	0.16 ± 0.09	0.24 ± 0.16
	L4 lower endplate		PS	±	±
			LES	0.27 ± 0.18	0.33 ± 0.19
			MF	0.24 ± 0.12	0.35 ± 0.21
	L5 lower endplate		PS	±	±
			LES	0.22 ± 0.09	0.37 ± 0.26
			MF	0.35 ± 0.14	0.49 ± 0.25

### Normalized fat-free muscle CSA (mCSA)

#### Multifidus

No significant interactions were found between the factors. The factor 'group' (p = 0.009) was significant. The mCSA was smaller in patients with pain compared to pain-free patients. The factor 'level of operation' (p = 0.796) was not significant. The factor 'slice' was significant (*p *< 0.001): the mean mCSA at the L3 slice was significantly smaller than on the L4 slice (*p *< 0.001) and on the L5 slice (*p *< 0.001); the mean mCSA on the L4 slice was significantly smaller than on the L5 slice (*p *< 0.001).

#### Lumbar erector spinae

Because a significant interaction was found between the factors 'group' and 'slice' (p = 0.049), comparisons between the two study groups were performed for each slice separately. Next, comparisons between slices were done for both patient groups. On the L3 slice, mCSA of the LES was significantly larger in pain-free patients than in pain patients (p = 0.016). On the L4 and L5 slice, no significant difference in mCSA of the LES was found between both patient groups.

Although a significant interaction was found for the factors 'group' and 'slice', the analysis for both groups sepperately revealed the same results:

the factor 'slice' was significant (*p *< 0.001); the mean mCSA at the L3 slice was significantly bigger than at the L4 slice (*p *< 0.001) and the L5 slice (*p *< 0.001); mean mCSA on the L4 slice was significantly larger than on the L5 slice (*p *< 0.001).

The factor 'level of operation ' was not significant (p = 0.638).

#### Psoas

Repeated measures analysis of variance showed no significant interactions between the main factors. The factor 'slice' was significant (*p *< 0.001). Post hoc analysis revealed a smaller mean mCSA on the L3 slice compared to the L4 slice (*p *< 0.001) and to the L5 (p = 0.001) slice. There was no significant difference in mCSA between the L4 and L5 slice (p = 0.398). The factors 'group' (p = 0.462) and 'level of operation' (p = 0.427) were not significant.

### Normalized fat CSA

#### Multifidus

No significant interactions were found between the factors. The factor 'slice' was significant (*p *< 0.001): the mean fCSA on the L3 slice was significantly smaller than on the L4 (*p *< 0.001) and the L5 slice (*p *= 0.001); the mean fCSA on the L4 slice was significantly smaller than on the L5 (p < 0.001). The factors 'group' (p = 0.186) and 'level of operation' (p = 0.146) were not significant.

#### Lumbar erector spinae

No significant interactions were found between the factors. The factors 'group' (p = 0.258), 'level of operation' (p = 0.131) and 'slice'(p = 0.208) were not significant.

#### Psoas

No significant interactions were found between the factors. The factor 'group' (p = 0.012) was significant. The fCSA was larger in patients with pain than in pain-free patients. The factor 'slice' was significant (*p *< 0.001): the mean fCSA at the L3 slice was significantly smaller than at the L4 slice (*p *= 0.001). There was no significant difference in fCSA between the L3 and the L5 slice (p = 0.054), nor between the L4 and L5 slice (p = 1.000). The factor 'level of operation' (p = 0.709) was not significant

## Discussion

### Pain-free patients compared to pain patients

#### MF and LES

##### Present findings

The results suggest atrophy of the MF at the three levels examined in the postoperative pain patients. Besides MF atrophy, LES atrophy was present in the pain patients at the L3 level only. Why LES atrophy was limited to the L3 level is fairly difficult to explain, especially since no surgical intervention was performed at this specific level and since there is no segmental innervation for the LES. It was hypothesized that the L4 and L5 levels also showed LES atrophy, but that muscle atrophy at these levels is more difficult to detect, because of the significantly smaller mCSA of the LES at the lower levels in all patients.

A few imaging studies have described back muscle atrophy in postoperative discectomy patients. Sihvonen et al. compared patients with good and poor results 2 to 5 years after surgery for spinal stenosis or disc herniation and found distinct back muscle atrophy in patients with poor results [14]. Mayer et al. reported a significantly lower back muscle density in spinal surgery patients compared to controls without back pain [13]. Motosuneya et al. studied back muscle atrophy in 5 lumbar surgery groups by measuring the CSA of the back muscles before and after surgery [18]. They documented a decrease in back muscle CSA in all groups, but the decrease was only significant in the lumbar fusion groups [18]. There was no significant difference in back atrophy regarding pain, as assessed using the Japanese Orthopaedic Association score for the management of LBP [18]. However, only 4 out of 49 patients reported frequent mild or occasional LBP [18].

##### Studies in nonoperative chronic LBP patients

Several studies have shown MF atrophy in chronic LBP patients [16-18,26]. Kader et al. visually analysed MRI images of LBP patients and reported MF atrophy in 80% of the patients [26]. The present imaging method is comparable to the one of Danneels et al., who compared chronic LBP patients with matched healthy subjects and found significant differences in muscle CSA only for the MF (limited to the L4 level), not for the PS and LES [17]. They therefore concluded that selective MF atrophy was present in chronic LBP [17]. In a study by Kamaz et al., muscle atrophy was especially prominent in the isolated MF, but also varying degrees of PA, quadratus lumborum and PS atrophy were found [11].

#### PS

The PS contained significantly more fat in the pain patients than in the asymptomatic subjects. However, the mCSA of the PS was not significantly different between the two groups. The present results suggest some PS deconditioning, without the presence of muscle atrophy. Previous data pertaining to PS size in postoperative patients have been conflicting. Mayer et al. found no significant difference in CSA of the PS in spinal surgery patients compared to controls without back pain [13]. In a study of predominantly surgical patients, Cooper et al. observed a significant decrease in muscle CSA of chronic LBP patients compared to acute LBP patients [12]. It is, however, unclear whether the results of the latter study applied to fat-free muscle CSA or total muscle CSA.

In the study by Kamaz et al., PS atrophy was documented in nonoperative chronic LBP patients [11]. Two studies of nonsurgical unilateral back pain compared CSA of the PS between the symptomatic and asymptomatic side. In nonsurgical LBP of at least 12 weeks' duration, Barker et al. found evidence of coexisting PS and MF atrophy (in terms of total muscle CSA) [16]. Dangaria et al. reported a significant decrease in ipsilateral psoas major CSA (total muscle CSA) in the presence of LBP and disc herniation, but could not conclude whether this was due to atrophy at the sciatic side or to hypertrophy at the other side [27]. In contrast, Danneels et al. observed no significant difference in PS mCSA and fCSA between chronic LBP patients and healthy volunteers [17].

### Level of operation

For none of the CSAs examined was the level of operation found to be a significant factor. Consequently, the operation did not seem to be responsible for the muscle atrophy observed. This finding is in accordance with the study by Sihvonen et al. In patients with poor recovery 2 to 5 years after surgery for spinal stenosis or disc herniation, they found muscle atrophy not to be restricted to the level of operation, but rather attributed it to muscle disuse [14]. Montesuneya et al. found back muscle atrophy to be present not only after posterior fusion, but also after anterior lumbar interbody fusion [18]. They concluded that besides direct surgical intervention, postoperative external fixation should also be held responsible for back muscle atrophy [18]. Also in this study, a weak positive correlation was documented between the atrophy ratio and the operating time only in posterior surgery, particularly nonfusion surgery. In a study by Kotilainen et al., the CSA of the lumbar muscles remained unchanged 6 months after microdiscectomy [28]. The authors attributed this to the tissue-sparing nature of the operation [28]. In the present study, MF deconditioning long after standard lumbar discectomy was not restricted to the level of operation, but was present on the 3 slices examined. In standard lumbar discectomy, the MF is only stripped off the spinous process and the vertebral arch at the level of operation. The exposure is minimal and the operating time is short, making muscle damage as the sole pain source in case of unsatisfactory results highly unlikely. However, the pain free patients had significantly more relief of back pain i*mmediately *postsurgery (p = 0.050), indicating that the cause of pain had been abolished during the operation. This was not entirely the case in the patients with persistent pain. The muscle abnormalities might therefore be the result and not the cause of their persistent pain. A more plausible explanation for MF atrophy at multiple levels and LES atrophy at the L3 level seems to be a pain-related inhibition phenomenon. Postoperative pain could have perpetuated the inhibition process that started at the occurrence of a symptomatic lumbar disc herniation. In the pain free patients, however, this was reversed by lumbar discectomy.

Because the TAMPA score for kinesiophobia was significantly higher in pain patients than in pain free patients, another hypothesis could be that back muscle deconditioning occurred as a consequence of fear avoidance in the patients with persistent pain. Back pain patients are more likely to avoid back extension movements than hip flexion movements, which explains why no atrophy was found for the mCSA of the PS. However, this muscle also showed signs of deconditioning as it contained more fat, which could be the result of some additive general muscle deconditioning in the pain patients.

### Limitations of the study

Since there are no data for healthy controls in the current study design, CT muscle quality of the pain free discectomy patients could not be compared with that of healthy controls. Questions remain concerning the presence of atrophy in pain free patients, which could for instance be influenced by the duration of pain preceding the disc surgery, or by back guarding to prevent recurrent LBP.

The clinical relevance of the differences between the pain group and the asymptomatic group still has to be proven in future studies.

A test-retest acquisition and consecutive measurements are necessary in order to estimate the reproducibility of the method. It was however not defendable to redo a 3D CT scan with 5000 Rad to this aim.

## Conclusions

Comparison of CT muscle condition of the MF, LES and PS in lumbar discectomy patients with pain and without pain long after surgery showed a smaller fat free muscle CSA of the MF at all levels examined, a smaller fat free muscle CSA of the LES at the L3 level, and more fat in the PS muscle in pain patients. The level of operation was not found to be of importance. The present results suggest a general lumbar muscle dysfunction in the pain group, and in particular of the deep stabilizing muscle system.

## Competing interests

The authors declare that they have no competing interests.

## Authors' contributions

KGWB participated in the study design, in collecting the data, the statistical analyses, and drafting of the manuscript. CT scan interpretation was performed by OV. VKS participated in collecting the data. PLC advised and assisted in the statistical analyses. CJJ and DCC participated in the study design. GGV coordinated the study. LAD participated in the study design and in the progress and drafting of the manuscript. All authors read and approved the final manuscript.

## Pre-publication history

The pre-publication history for this paper can be accessed here:

http://www.biomedcentral.com/1471-2474/12/65/prepub
